# Transmissible Gastroenteritis Virus Infection Enhances SGLT1 and GLUT2 Expression to Increase Glucose Uptake

**DOI:** 10.1371/journal.pone.0165585

**Published:** 2016-11-16

**Authors:** Lei Dai, Wei Wei Hu, Lu Xia, Mi Xia, Qian Yang

**Affiliations:** Veterinary College, Nanjing Agricultural University, Weigang 1, Nanjing, Jiangsu, PR China; Deutsches Primatenzentrum GmbH–Leibniz-Institut fur Primatenforschung, GERMANY

## Abstract

Transmissible gastroenteritis virus (TGEV) is a coronavirus that causes villus atrophy, followed by crypt hyperplasia, reduces the activities of intestinal digestive enzymes, and disrupts the absorption of intestinal nutrients. *In vivo*, TGEV primarily targets and infects intestinal epithelial cells, which play an important role in glucose absorption via the apical and basolateral transporters Na^+^-dependent glucose transporter 1 (SGLT1) and facilitative glucose transporter 2 (GLUT2), respectively. In this study, we therefore sought to evaluate the effects of TGEV infection on glucose uptake and SGLT1 and GLUT2 expression. Our data demonstrate that infection with TGEV resulted in increased glucose uptake and augmented expression of EGFR, SGLT1 and GLUT2. Moreover, inhibition studies showed that EGFR modulated glucose uptake in control and TGEV infected cells. Finally, high glucose absorption was subsequently found to promote TGEV replication.

## Introduction

Transmissible gastroenteritis (TGE) is a highly contagious infectious disease of pigs that is characterized by vomiting, diarrhea, and dehydration. Notably, the mortality rate of this disease in seronegative suckling piglets can reach up to 100% [1]. TGE is caused by the TGE virus (TGEV), which infects the gastrointestinal tract, causing villus atrophy and crypt hyperplasia, and disrupting intestinal nutrition absorption [2, 3].

Glucose is among the nutrients absorbed in the intestinal epithelium, and glucose uptake in epithelial cells depends on two types of glucose transporters, the apically expressed Na^+^-dependent glucose transporter 1 (SGLT1) and basolaterally expressed facilitative glucose transporter 2 (GLUT2). Specifically, SGLT1 mediates the Na+/glucose cotransport function of the kidney and intestine as a secondary active transporter, while GLUT2 serves as a facilitated diffusion system for transport through lipid bilayers [4–6]. The epidermal growth factor receptor (EGFR) was previously reported to transiently increase glucose transport [7, 8]. Moreover, a recently study suggested that EGFR may act as another receptor for TGEV, in addition to porcine aminopeptidase (pAPN) [9]. EGFR-dependent regulation of glucose uptake has been observed in tumor cells, and EGFR has been shown to prevent autophagic cell death by maintaining intracellular glucose levels through interaction with and stabilization of SGLT1 [10]. However, the involvement of EGFR in virus-induced effects on glucose uptake has yet to be evaluated.

Therefore, in the study, we aimed to examine the *in vitro* effects of TGEV infection on glucose uptake and the expression of SGLT1 and GLUT2 in porcine intestinal columnar epithelial (IPEC-J2) cells, which have been shown to offer a practical model for studying TGEV infection [11, 12].

## Materials and Methods

### Cell lines

IPEC-J2 cells, which are porcine intestinal columnar epithelial cells that were originally isolated from the middle jejunum of neonatal piglets, were purchased from DSMZ (Braunschweig, Germany), while HEK293T cells were purchased from the American Type Culture Collection (ATCC; Manassas, VA, USA). IPEC-J2 and HEK293T cells were maintained in Roswell Park Memorial Institute medium (RPMI) and Dulbecco’s modified Eagle’s medium (DMEM) with high glucose, respectively, supplemented with HEPES, 10% fetal bovine serum (FBS; Gibco, Grand Island, NY, USA), and 1% penicillin-streptomycin (Invitrogen, Carlsbad, CA, USA) at 37°C in a 5% CO_**2**_ incubator (Thermo Fisher Scientific, Waltham, MA, USA).

### Viral infection

TGEV strain SHXB was isolated in Shanghai, China. The complete genome sequence for this virus is available at the GenBank database (ID number: KP202848.1) [13]. For experimental assays, cells were incubated with TGEV at a multiplicity of infection (MOI) of 2 for 1 h at 4°C in serum-free medium and washed with phosphate-buffered saline (PBS; pH 7.2) at 4°C three times to remove unbound virus. Cells were then cultured in medium containing 2% serum.

### RNA extraction and reverse transcription polymerase chain reaction (RT-PCR)

Total RNA was extracted from IPEC-J2 cells infected with TGEV using TRIzol reagent (Invitrogen), according to the manufacturer’s instructions. cDNA was generated by reverse transcription using HiScript QRT SuperMix for qPCR (Vazyme Biotech, Nanjing, China), according to the manufacturer’s instructions. TGEV release was assessed by measuring the levels of viral nucleoprotein (N) gene expression via quantitative RT-PCR using a TaKaRa SYBR Green qPCR Kit (TaKaRa, Shiga, Japan). The primer sequences were as follows: N-F (sense), 5'-CAATTCCCGTGGTC GGAAGA-3', N-R (antisense), 5'-TTTACGTTGGCCCTTCACCA-3'. PCR products were purified using a Gel Extraction Kit (Omega Bio-Tek, Inc., Norcross, GA, USA) and cloned into the pJET1.2 vector (Thermo Fisher). Plasmids were serially diluted and used as standards for quantitative analysis. The initial copy number of the TGEV N gene was calculated using the following formula: X0 = -K log Ct + b, where X0 is the initial copy number and K, Ct, and b refer to the slope rate, cycle threshold, and constant, respectively. Quantitative real-time PCR was performed with an ABI PRISM 7500 Detection System (Applied Biosystems, Foster City, CA, USA).

### Western blotting

At the indicated time points post-infection, cells were washed with PBS and lysed in radioimmunoprecipitation assay (RIPA) buffer (Thermo Scientific) containing a phosphatase inhibitor and protease inhibitor (Thermo Scientific), according to the manufacturer’s instructions. The protein concentrations of the resulting lysates were determined using a Pierce BCA Protein Assay kit based on the bicinchoninic acid spectrophotometric method (Thermo Scientific). After centrifugation at 13,000 × *g* for 15 min, proteins in the supernatant (15–50 μg protein) were separated by sodium dodecyl sulfate polyacrylamide gel electrophoresis (SDS-PAGE) on 10–12% gradient gels, and transferred to polyvinylidene fluoride (PVDF) membranes (Merck Millipore, Darmstadt, Germany). Membranes were blocked for 2 h in Tris-buffered saline (TBS) containing 5% nonfat dry milk, and probed with the indicated primary antibodies at 4°C overnight, according to the manufacturer’s instructions. The following antibodies were used in this study: anti-p-EGFR antibody (D7A5; Cell Signaling Technology, Danvers, MA, USA), anti-EGFR (c4267; Cell Signaling Technology, Danvers, MA, USA), anti-SGLT1 (ab14686; Abcam, Cambridge, UK), anti-GLUT2 (sc-7580; Santa Cruz Biotechnology, Dallas, TX, USA), anti-β-tubulin (E12-043; Enogene Biotech, New York, NY, USA). Membranes were then exposed to species-specific horseradish peroxidase (HRP)-conjugated secondary antibodies (dilution: 1:5,000), and proteins were detected by enhanced chemiluminescence (ECL; Thermo Scientific) and autoradiography. The resulting bands were quantified using Quantity One 1-D Analysis Software (170–9600; Bio-Rad, Hercules, CA, USA). The density of each band was measured and normalized to that of β-tubulin expression. All data were expressed as means ± standard deviations (SD) of the results of three independent experiments.

### Lentivirus-mediated RNA interference (RNAi) depletion experiments

pLVX-shRNA is a human immunodeficiency virus type 1 (HIV-1)-based lentiviral expression vector designed to express a small hairpin RNA (shRNA) for RNAi studies (Clontech Laboratories, Inc., Mountain View, CA, USA). The best silencing efficiencies were observed with clones NM_214007 (porcine EGFR) and NM-001012297.1 (porcine SGLT1). The shRNA for EGFR, three shRNAs for SGLT1, and overexpression plasmid for EGFR were designated as shEGFR, shSGLT1-1, shSGLT1-2, shSGLT1-3, and pLVX-EGFR, respectively. HEK293T cells were transfected with 1 μg of specific expression plasmid per 10^6^ cells using the X-tremeGENE HP DNA Transfection Reagent (Roche, Basel, Switzerland), according to the manufacturer’s instructions, diluted in Opti-MEM (Invitrogen) in a T-25 cell culture flasks. Lentiviral particles (MOI = 2) were subsequently added to the transfected IPEC-J2 cells and gently mixed.

### Inhibitors

After comparing amino acid sequences of EGFR, SGLT1 and GLUT2 from NCBI (Table 1), we respectively selected AG1478, phlorizin as the inhibitor of EGFR and SGLT1.

**Table 1:** Comparison of amino acid sequences.

*Definition*	*Sus scrofa NCBI sequence*	*Homo sapiens NCBI sequence*	*Amino acid similarities*
*EGFR*	*NM_214007*	*CAA25240*.*1*	*88%*
*SGLT1*	*NM-001012297*.*1*	*NP_001035915*.*1*	*88%*
*GLUT2*	*AMN88560*.*1*	*NP_000331*.*1*	*87%*

### Glucose uptake experiments

2-[^3^H]deoxyglucose (2-DG) uptake experiments were carried out according to the method described by Henriksen *et al*., but with modifications [14]. Briefly, the culture medium was removed by aspiration, and cells were gently washed twice with uptake buffer (140 mM NaCl, 2 mM KCl, 1 mM KH_2_PO_4_, 10 mM MgCl_2_, 1 mM CaCl_2_, 5 mM glucose, 5 mM l-alanine, 5 nM indomethacin, and 10 mM HEPES-Tris; pH 7.4). After washing, cells were incubated in uptake buffer containing 2-DG at 37°C for 30 min. At the end of the incubation period, cells were washed three times with ice-cold uptake buffer and dissolved in 1 mL of 0.1% SDS. The level of intracellular 2-DG uptake was determined by measuring the radioactivity of a 900-μL aliquot of each sample using a liquid scintillation counter (LS 6500; Beckman Instruments, Fullerton, CA, USA). The remainder of each sample was then used to determine protein expression levels of glucose transporters. The radioactivity counts of each sample were normalized with respect to the protein and corrected for time uptake per milligram of protein. All uptake measurements were carried out in triplicate. Glucose uptake was calculated as follows: glucose uptake (nM/10^6^ cells) = (glucose concentration of the control group–glucose concentration of the experimental group)/cell number.

### Carboxyfluorescein succinimidyl ester (CFSE) labeling

CFSE stocks (10 mM in DMSO; Invitrogen, Merelbeke, Belgium) were stored at -20°C prior to use, thawed, and diluted in phosphate-buffered saline (PBS) to the desired working concentrations. IPEC-J2 cells were resuspended in PBS (0.1% BSA) at 2 × 10^6^ cells/mL and incubated with CFSE (final concentration: 1 μM) for 7 min at 37°C. Cells were washed and resuspended in culture medium for 15 min to stabilize CFSE staining, and CFSE-labeled cells were analyzed by flow cytometry.

### Cell counting kit-8 assays

The viability of RAW264.7 cells was determined using a Cell Counting Kit-8 Assay Kit (Beyotime Biotechnology, Beijing, China), as previously reported. Briefly, RAW264.7 cells were plated at a density of 1 × 10^4^ cells/well in 96-well plates in 100 mL Roswell Park Memorial Institute 1640 medium and incubated for 24 h. Twenty microliters of cell counting kit-8 reagent was then added to each microwell, and plates were incubated for 2 h at 37°C. The absorbance of the colored solution was measured using a microplate reader (Bio-Rad Laboratories) at a test wavelength of 450 nm and a reference wavelength of 630 nm.

### Statistical analysis

All results are presented as means ± SD of the results of three independent experiments. Significant differences between control and experimental groups were analyzed using Student’s *t*-test. *p*-values < 0.05 were considered statistically significant.

## Results

### TGEV infection results in increased glucose uptake

**Fig 1:**
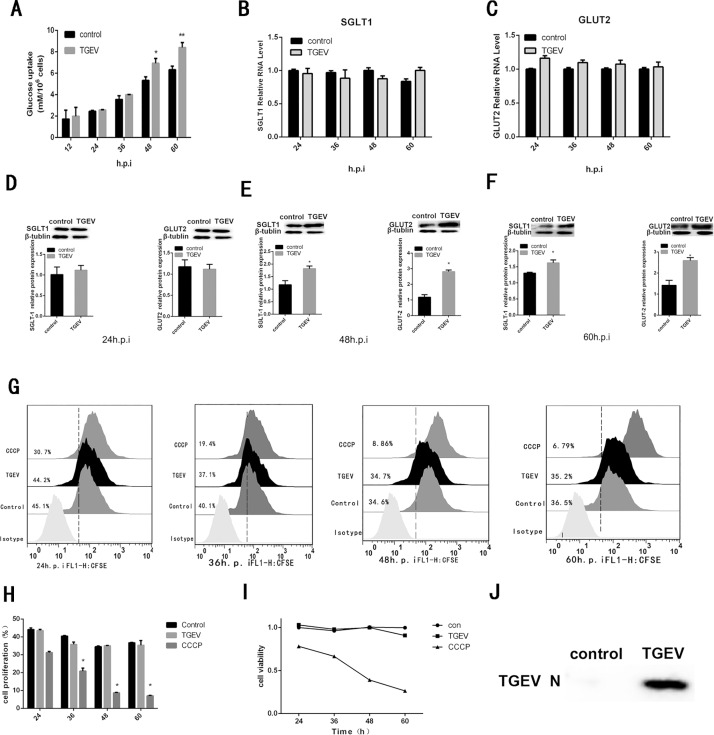
TGEV infection promotes glucose uptake via increased protein expression of SGLT1 and GLUT2. (A) IPEC-J2 cells were incubated with TGEV (MOI = 2) at 4°C for 1 h and then at 37°C. The culture medium was then collected at the indicated time points, and the glucose concentration in the medium was assayed. Total RNA was isolated at 24, 48 and 60 hours post-infection (hpi). (B) *SGLT1* and (C) *GLUT2* (C) mRNA expression levels were measured by quantitative RT-PCR and normalized to that of the *PPIA* gene. (D–E) Whole-cell extracts were prepared from mock-infected (control) and TGEV-infected (TGEV) IPEC-J2 cells at (D) 24, (E) 48, and (F) 60 hpi. (J) The protein expression levels of SGLT1, GLUT2, β-tubulin, and TGEV N protein were determined by western blot analysis. (G) Carboxyfluorescein succinimidyl ester (CFSE) was used to label mock-infected (control), TGEV-infected (TGEV), and carbonyl cyanide *m*-chlorophenyl hydrazone (CCCP)-treated (CCCP; 10 mM) cells, and flow cytometric analyses was performed at 24, 48, and 60 hpi. (H) Data were quantified using SPSS software. (I) CCK-8 assays were performed to quantitatively assess the viability of cells incubated in DMEM supplemented with 10% FBS. All CCK-8 assays were performed using six parallel samples. Statistical significance was assessed by Student’s t-tests. Differences were considered significant at (*) 0.01 < *p* < 0.05 or (**) *p* < 0.01. All experiments were performed separately three times.

As shown as in Fig 1A, TGEV infection triggered a significant increase in glucose uptake in IPEC-J2 cells at 48 and 60 h post-infection (hpi). In addition, during late-stage infection, TGEV-infected cells were able to continue transporting glucose, even when the concentration of glucose in the cell culture medium was low. These data indicate that TGEV infection promotes glucose uptake in IPEC-J2 cells.

While Fig 1B–1F show the mRNA and protein levels of SGLT1 and GLUT2 at various time points. Notably, while there were no marked changes in the mRNA expression of the *SGLT1* and *GLUT2* genes after TGEV infection, there were significant increases in the protein expression of both SGLT1 and GLUT2 at 48 and 60 hpi. To exclude the possibility that cell viability and proliferation may affect glucose uptake, CFSE-labeled mock-infected (control), TGEV-infected (TGEV), and carbonyl cyanide *m*-chlorophenyl hydrazone (CCCP)-treated (CCCP) cells were subjected to flow cytometric analysis. CCCP is known to reduce cell viability and proliferation, and was therefore utilized as a positive control [15]. As shown in Fig 1G–1I, compared with CCCP treatment, TGEV infection had no significant effect on the proliferation and viability of IPEC-J2 cells. Fig 1J shows TGEV infection in IPEC-J2 cells at 24hpi.

### TGEV infection increases EGFR protein expression

**Fig 2:**
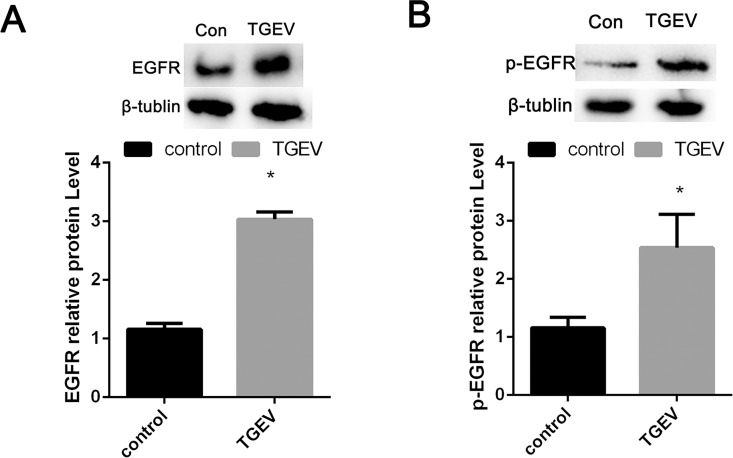
**TGEV infection increased EGFR expression** Western blot analysis was performed to determine the levels of EGFR (A) and p-EGFR (B) in mock-infected (control) or TGEV-infected (TGEV) cell extracts harvested at 48 hpi. β-tubulin was also analyzed.

Previous studies have shown that the expression of EGFR is closely related to glucose transport, especially phosphorylation region of EGFR. EGFR kinase activity regulates the peak glucose uptake and total EGFR may likely be maintaining basal glucose uptake [7, 8]. Therefore, we first examined whether EGFR expression was stimulated by TGEV infection. Western blot analysis showed that TGEV infection not only increased total EGFR expression (Fig 2A) but also revealed that more phosphorylated EGFR was present in TGEV infected relative to control cells (Fig 2B). Taken together, these results indicated that TGEV infection increased the total and phosphorylation protein expression of EGFR at 48 hpi. Together, these results indicate that TGEV infection promoted the total and phosphorylation protein expression of EGFR at 48 hpi.

### EGFR regulates glucose uptake via interaction with SGLT1 in mock-infected cells

**Fig 3:**
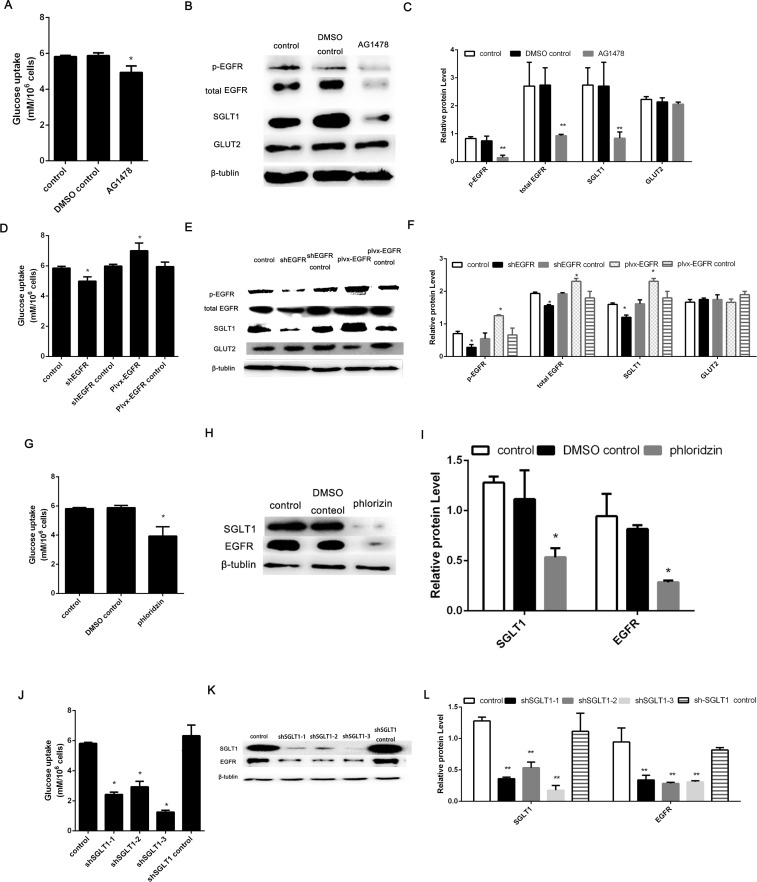
EGFR influences glucose uptake levels in mock-infected IPEC-J2 cells, and the expression of EGFR and SGLT1 protein was dynamic. (A) Cells were treated with PBS, DMSO (100 nM), or AG1478 (100 nM) at 37°C for 48 h. The culture medium from each group was then assayed. (B) Western blot analysis of the expression levels of p-EGFR, EGFR, SGLT1, GLUT2, and β-tubulin. Experiments were performed separately three times. (C) The intensities of protein bands were quantified using Quantity One and SPSS software. (D) Cells were transfected with shEGFR, shEGFR control, pLVX-EGFR, or pLVX-EGFR control. After 48 h of incubation, culture medium from each group and from mock-infected cells was assayed. (E) The expression levels of p-EGFR, EGFR, SGLT1, GLUT2, and β-tubulin proteins were evaluated by western blot analysis. Experiments were performed separately three times. (F) The intensities of protein bands were quantified using Quantity One and SPSS software. (G) IPEC-J2 cells were treated with phlorizin (500 μM) at 37°C for 48 h, and culture medium from each group was assayed. (H) The expression levels of SGLT1, EGFR, and β-tubulin were evaluated by western blot analysis. (I) The intensities of protein bands were quantified using Quantity One and SPSS software. (J) IPEC-J2 cells were treated with three SGLT1-specific siRNA molecules for 48 h, and culture medium from each group was assayed. (K) Western blot analysis of the expression levels of SGLT1, EGFR, and β-tubulin. (L) The intensities of protein bands were quantified using Quantity One and SPSS software. Each experiment was performed separately three times.

To explore whether EGFR contributes to the observed TGEV infection increase in glucose uptake, we explored changes in glucose uptake and the expression of glucose transport molecules by inhibiting or overexpressing EGFR in mock-infected cells. We choose AG1478 as EGFR inhibition because it can inhibit the EGFR tyrosine kinase activity and total EGFR [16, 17]. As shown in Fig 3A–3C, treatment with the EGFR inhibitor AG1478 suppressed glucose uptake and markedly decreased EGFR and p-EGFR protein expression, indicating that AG1478 regulated glucose uptake in mocked-infected cells. Furthermore, cells treated with AG1478 exhibited markedly lower levels of SGLT1 protein expression than the control cells; in contrast, there was no effect on GLUT2 protein expression. Thus, we concluded that AG1478 modulated glucose uptake in mock-infected cells via downregulation of EGFR, which resulted in reduced SGLT1 expression.

As shown in Fig 3D–3F, we sought to then strengthen these findings via modulation of EGFR expression. Transfection with shEGFR resulted in significant downregulation of EGFR and p-EGFR protein expression and decreased glucose uptake compared with the cells transfected with the control shRNA. Conversely, pLVX-EGFR transfection resulted in significantly increased EGFR and p-EGFR protein expression and glucose uptake. These data demonstrate that modulation of EGFR and p-EGFR expression affects glucose uptake in the absence of TGEV infection. Furthermore, transfection with shEGFR and pLVX-EGFR resulted in decreased and increased SGLT1 protein expression, respectively; in contrast, GLUT2 protein expression was unaffected by either treatment. Together, these results indicate that EGFR and p-EGFR regulates glucose uptake in mock-infected IPEC-J2 cells by modulation of SGLT1 protein expression.

Because SGLT1 is involved in mediating the functions of EGFR in HEK-293T cells [18], we explored the relationship between EGFR and SGLT1 expression in IPEC-J2 cells. As shown in Fig 3H and 3J–3L, IPEC-J2 cells transfected with three SGLT1-specific shRNAs for 48 h exhibited reduced SGLT1 expression and reduced EGFR protein expression. Furthermore, we choose phlorizin as SGLT1 inhibitor because phlorizin's principal pharmacological action is to block intestinal glucose absorption through inhibition of the sodium–glucose symporters located inmucosa of the small intestine [19, 20]. IPEC-J2 cells treated with phlorizin for 48 h showed downregulation of SGLT1 expression without inducing cell death, as demonstrated by CCK8 assay analyses (data not shown). Similarly, western blot analysis showed that IPEC-J2 cells treated with phlorizin exhibited lower EGFR expression than DMSO-treated cells. In conclusion, inhibition of SGLT1 by shRNA or phlorizin treatment disrupted EGFR expression. Notably, these treatments also resulted in decreased glucose uptake (Fig 3G and 3J). Consistent with these findings, IPEC-J2 cells transfected with shEGFR or treated with AG1478 also exhibited lower SGLT1 expression (Fig 3B and 3E). These data indicate that there is an association between EGFR and SGLT1 expression in IPEC-J2 cells.

### TGEV infection-activated EGFR results in increased glucose uptake

**Fig 4:**
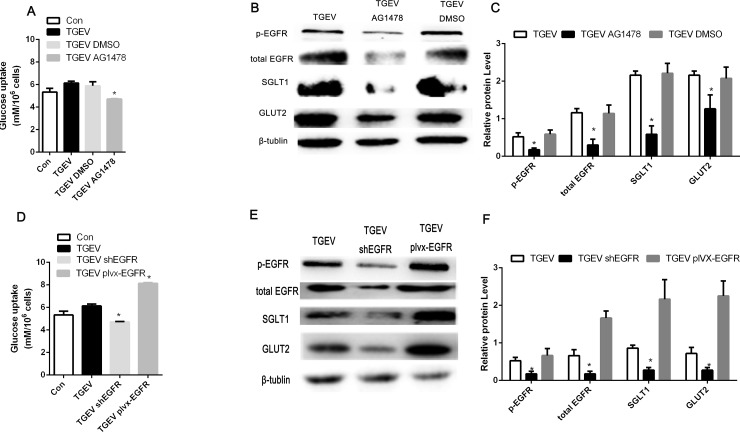
EGFR influences glucose uptake in TGEV-infected IPEC-J2 cells. (A) Cells were treated with PBS, DMSO (100 nM), or AG1478 (100 nM) at 37°C for 48 h. At 48 h after TGEV infection, culture medium from each group was assayed. (B) Western blot analysis of p-EGFR, EGFR, SGLT1, GLUT2, and β-tubulin protein expression. (C) The intensities of protein bands were quantified using Quantity One and SPSS software. (D) Cells were transfected with shEGFR or pLVX-EGFR for 48 h. At 48 h after TGEV infection, culture medium from each group and from mock-infected cells was assayed. (E) The protein expression of p-EGFR, EGFR, SGLT1, GLUT2, and β-tubulin was determined by western blot analysis. Experiments were performed separately three times. (F) The intensities of protein bands were quantified using Quantity One and SPSS software. Each experiment was performed separately three times.

To further investigate the role of TGEV infection-enhanced EGFR in the regulation of glucose uptake, we examined the effects of TGEV infection after treatment or transfection with AG1478, shEGFR, or pLVX-EGFR on glucose uptake. As shown in Fig 4A and 4D, transfection with shEGFR and treatment with AG1478 significantly inhibited glucose uptake, whereas transfection with pLVX-EGFR enhanced glucose uptake. Therefore, we concluded that EGFR mediates glucose uptake in TGEV-infected IPEC-J2 cells. Moreover, As shown in Fig 4B, 4C, 4E and 4F, after TGEV infection, western blot analysis showed that transfection with shEGFR and treatment with AG1478 resulted in lower protein expression of SGLT1 and GLUT2 than that observed in the control group, whereas transfection with pLVX-EGFR resulted in increased SGLT1 and GLUT2 protein expression. Together, these results indicate that EGFR influences glucose uptake in TGEV-infected cells by promoting both SGLT1 and GLUT2 expression.

### TGEV replication are affected by intracellular glucose concentrations

**Fig 5:**
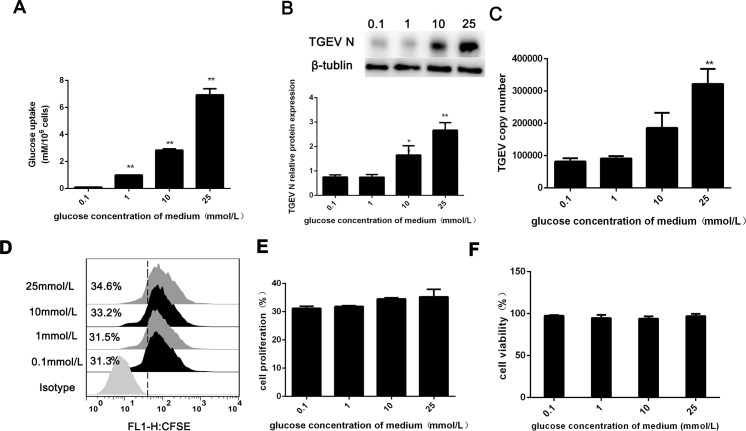
Glucose promotes TGEV replication. (A) IPEC-J2 cells were cultured in medium containing 0.1, 1, 10, or 25 mM glucose. Culture medium was then assayed. (B) Whole-cell extracts were prepared after TGEV infection for 48 h, and samples were analyzed for TGEV N and β-tubulin protein expression by western blotting. (C) Total RNA was extracted from culture medium supernatants, and TGEV copy numbers were determined by RT-PCR analysis. (D) Flow cytometric analysis of cells cultured in medium containing 0.1, 1, 10, or 25 mM glucose and labeled with carboxyfluorescein succinimidyl ester (CFSE). (E) The resulting data were quantified using SPSS software. (F) CCK-8 assays were performed to quantitatively assess the viability of cells. All CCK-8 assays were performed using six parallel samples.

Previous studies have suggested that the intracellular glucose concentration is closely linked with viral infections, particularly for double-stranded DNA viruses [21–23]. Consistent with this conclusion, we found that TGEV enhanced glucose uptake in IPEC-J2 cells. Because glucose is the main energy source for cellular metabolism, TGEV replication in IPEC-J2 cells requires large amounts of glucose/energy. Thus, we explored whether high glucose uptake in TGEV-infected cells affected TGEV replication. TGEV contains a 27.6–31.3-kb single-stranded, positive-sense RNA genome; the virion RNA functions as an mRNA and is infectious. The N protein, in particular, is a critical component of the replication-transcription complex [24–26]. Thus, we treated TGEV-infected cells with medium containing 0.1, 1, 10, or 25 mM glucose and harvested the virus at 48 hpi. As shown in Fig 5A, glucose absorption concentrations were 0.1, 1, 1.8, and 5.2 mM for medium containing 0.1, 1, 10, and 25 mM glucose, respectively. Furthermore, western blot analysis showed that TGEV N protein was more abundant when TGEV-infected cells absorbed more glucose, particularly in medium containing 10 or 25 mM glucose (Fig 5B). Consistent with this finding, there was a concurrent increase in the copy number of the TGEV N gene as glucose absorption levels increased (Fig 5C). As described above, in TGEV-infected IPEC-J2 cells, higher glucose concentrations promoted TGEV replication. To exclude the possibility that cell viability and proliferation affect TGEV replication, flow cytometric analysis of CFSE-labeled cells was performed in the presence of 0.1, 1, 10, and 25 mM glucose. As shown in Fig 5D–5F, high glucose concentrations did not significantly affect proliferation and viability.

## Discussion

As recently reported, viral infection may result in increased or decreased glucose uptake. For example, the hepatitis B virus X protein regulates hepatic glucose homeostasis via nitric oxide synthase [27, 28]. Additionally, human cytomegalovirus (HCMV) enhances glucose uptake during the first 20 h after infection, even when a high level of glucose is constantly present in the medium [22]. Hepatitis C virus (HCV) replication suppresses cellular glucose uptake through downregulation of cell surface glucose transporters [29], while rotavirus infection impairs rabbit intestinal brush-border membrane Na^+^-solute cotransport activities [30]. However, the effects of coronaviruses (including TGEV) on glucose uptake have not been reported. we found that TGEV infection increased glucose uptake of IPEC-J2 cells and the protein expression of SGLT1, GLUT2 after 48 hpi (Fig 1), while TGEV infection in IPEC-J2 cells reached a peak at 48 hpi [31], we just provided foresight to explore the interaction between TGEV infection and glucose uptake, which is still worth exploring.

Most of the glucose absorbed in the intestine is transformed to glutamine [32], which was reported to promote the replication of many viruses, such as porcine circovirus (PCV) and HCMV, *in vitro* [33, 34]. Because viral replication requires a lot of energy, we examined the effects of glucose absorption on TGEV propagation. Our results demonstrate that increased glucose absorption enhanced TGEV replication, suggesting that glucose may be transformed into glutamine in infected cells. In this study, increases in SGLT1 and GLUT2 protein expression directly stimulated glucose uptake *in vitro*. Although the role of other transporters cannot be ruled out, as SGLT1 and GLUT2 dominate intestinal glucose transport, they may play important effect on glucose uptake in TGEV infection. However, no significant changes were observed in SGLT1 or GLUT2 mRNA expression levels, indicating that TGEV infection primarily affects the protein expression of SGLT1 and GLUT2. These findings were consistent with previous reports demonstrating that TGEV infection influences protein translation. Additionally, SGLT1 and GLUT2 have been shown to mediate intestinal glucose uptake through *de novo* synthesis of mRNA and protein [35]. Within the last decade, the mechanisms of passive or diffusive components of intestinal glucose uptake have come under debate [36]. The current paradigm describing intestinal uptake is that glucose enters the absorptive cell through SGLT1 in the brush-border membrane and exits into the blood through GLUT2, a member of the facilitative glucose transporter family, located in the basolateral membrane. We have now provided evidence for an alternative mechanism for the passive component of absorption, i.e., rapid trafficking of GLUT2 to the brush-border membrane was found to be controlled by the SGLT1-dependent activation of a protein kinase C (PKC)-dependent pathway and also by mitogen-activated protein kinase (MAPK) intracellular signaling pathways [37–39]. GLUT2 exhibits a >10-fold-higher glucose transport capacity than SGLT1 and provides a major route of glucose absorption with rapid absorptive capacity [40, 41]. GLUT2 plays an important role in glucose absorption across the brush border membrane in normal jejunum [42]. Although our experiments did not directly demonstrate the rapid trafficking of GLUT2, we found that TGEV infection significantly increased glucose uptake at 48 h, indicating accelerated glucose absorption. Thus, TGEV infection might promote glucose trafficking through GLUT2. Moreover, because the TGEV increased the protein expression of p-EGFR (Fig 2B) and TGEV spike protein is capable of binding to EGFR, thereby activating the downstream PKC-dependent and MAPK intracellular signaling pathways [9], the trafficking of GLUT2 to the brush-border membrane could be stimulated, allowing rapid transport of glucose.

Previous studies have shown that glucose transport plays an important role in viral invasion. GLUT1-mediated glucose transport regulates HIV infection [43] and is a receptor for human T-cell leukemia virus (HTLV). Additionally, perturbations in glucose metabolism resulting from interactions between HTLV envelope proteins and GLUT1 are likely to contribute to HTLV-associated disorders [44]; thus, analysis of the relationship between viral infection and glucose uptake is critical. Notably, in mock-infected cells, EGFR mediate SGLT1 protein expression. Indeed, previous literatures reported that EGFR maintains intracellular glucose levels through interaction with and stabilization of SGLT1 [10] [18]. Importantly, we found that EGFR modulated SGLT1 expression during infection, but it cannot be concluded unfortunately that the cellular effect of TGEV infection on SGLT1 is mediated via EGFR. Because the drug treatment and knock down/overexpression against SGLT1 had similar effects with EGFR (Figs 3 and 4). In other words, it could also be direct effect on SGLT1, which in results in similar effect on EGFR. In fact, EGFR and SGLT1 are co-expressed in many other type cells [45, 46]. Moreover, previous studies have shown that EGFR is a target receptor for viruses and bacteria, including TGEV and *Escherichia coli* [9, 47]. Thus, because EGFR also interacts with SGLT1, this protein might comprise another ubiquitous target receptor.

EGFR has been reported to increased glucose uptake which is critical for the cell [10], we found the EGFR and p-EGFR both promote glucose uptake in IPEC-J2 cells during TGEV infection. Previous literatures reported that EGFR kinase activity regulates the peak glucose uptake and total EGFR may likely be maintaining basal glucose uptake [7, 8]. In our study, the IPEC-J2 cells have been in a rich glucose medium during TGEV infection, we also did not explore the respective role of EGFR and p-EGFR in glucose uptake.

The mechanism through which TGEV causes diarrhea has not been elucidated. On the other hand, glucose uptake has been shown to cause diarrhea [48, 49]. For example, enteropathogenic *E*. *coli* rapidly inactivates SGLT1 through multiple mechanisms. Indeed, the finding that one mechanism occurs more rapidly than microvilli effacement provides a plausible explanation for the rapid onset of severe watery diarrhea, given the crucial role of SGLT1 in the daily uptake of large amounts of fluids from the normal intestine. In contrast, our data indicate that TGEV infection resulted in increased SGLT1 expression. However, TGEV infection did lead to rapid glucose uptake, which in turn supplied glucose to the virus and promoted long-term infection by TGEV in the intestine.
